# Severe acute respiratory syndrome-coronavirus infection in aged nonhuman primates is associated with modulated pulmonary and systemic immune responses

**DOI:** 10.1186/1742-4933-11-4

**Published:** 2014-03-19

**Authors:** Candice C Clay, Nathan Donart, Ndingsa Fomukong, Jennifer B Knight, Katie Overheim, Jennifer Tipper, Jesse Van Westrienen, Fletcher Hahn, Kevin S Harrod

**Affiliations:** 1 Work performed at Lovelace Respiratory Research Institute (LRRI), Infectious Disease Program, Albuquerque, NM, Mexico; 2 California National Primate Research Center, University of California, Davis, CA, USA

## Abstract

**Background:**

Many respiratory viruses disproportionately impact the elderly. Likewise, advanced age correlated with more adverse disease outcomes following severe acute respiratory syndrome coronavirus (SARS-CoV) infection in humans. We used an aged African green monkey SARS-CoV infection model to better understand age-related mechanisms of increased susceptibility to viral respiratory infections. Nonhuman primates are critical translational models for such research given their similarities to humans in immune-ageing as well as lung structure.

**Results:**

Significant age- and infection-dependent differences were observed in both systemic and mucosal immune compartments. Peripheral lymphocytes, specifically CD8 T and B cells were significantly lower in aged monkeys pre- and post- SARS-CoV infection, while neutrophil and monocyte numbers were not impacted by age or infection status. Serum proinflammatory cytokines were similar in both age groups, whereas significantly lower levels of IL-1beta, IL-18, IL-6, IL-12 and IL-15 were detected in the lungs of SARS-CoV-infected aged monkeys at either 5 or 10 days post infection. Total lung leukocyte numbers and relative frequency of CD8 T cells, B cells, macrophages and dendritic cells were greatly reduced in the aged host during SARS-CoV infection, despite high levels of chemoattractants for many of these cells in the aged lung. Dendritic cells and monocytes/macrophages showed age-dependent differences in activation and chemokine receptor profiles, while the CD8 T cell and B cell responses were significantly reduced in the aged host. In examination of viral titers, significantly higher levels of SARS-CoV were detected in the nasal swabs early, at day 1 post infection, in aged as compared to juvenile monkeys, but virus levels were only slightly higher in aged animals by day 3. Although there was a trend of higher titers in respiratory tissues at day 5 post infection, this did not reach statistical significance and virus was cleared from all animals by day 10, regardless of age.

**Conclusions:**

This study provides unique insight into how several parameters of the systemic and mucosal immune response to SARS-CoV infection are significantly modulated by age. These immune differences may contribute to deficient immune function and the observed trend of higher SARS-CoV replication in aged nonhuman primates.

## Introduction

Viral respiratory infections remain a predominant cause of morbidity and mortality in aged adults. The elderly have heightened susceptibility to infection, an increased risk of developing severe viral-induced pulmonary disease and have slower recovery rates [1]. Several physiological parameters are thought to contribute to the poor outcomes of infectious disease in the elderly population, including the aging immune as well as respiratory system. Almost all components of the immune system have been shown to undergo age-associated restructuring that greatly impacts immune function [2-5]. The decline in immune function with age also results in reduced vaccine efficacy, further enhancing susceptibility to infection in the elderly [6-8].

Age-associated alterations in the mucosal immune system are thought to occur at distinct times and in a distinct manner relative to systemic immunity [9]. Data suggests that immunosenescence may occur earlier in the mucosa than the systemic immune system with a dramatic shift, with age, in the proportion of distinct T cell subsets and a decrease in total B lymphocytes [3,10]. Advanced age has also been associated with a reduction in antigen-specific IgA, an important protective antibody predominantly localized to the mucosa [9]. In addition to the immunological remodeling as a function of age, there are also major alterations in respiratory physiology. The aging lung has been shown to undergo structural changes which include a loss in static recoil forces, a stiffening of the chest cavity and diminished alveolar surface area, ultimately resulting in reduced vital capacity [11-13]. In addition, respiratory muscle strength consistently declines with age making it more difficult for an elderly person to breath even when not suffering from a respiratory infection.

The limitations of the aged immune and respiratory systems likely contributed to the increased mortality observed in elderly patients (>60 years old) with severe acute respiratory syndrome coronavirus (SARS-CoV). The SARS-CoV epidemic in 2002–2003 resulted in over 8000 human infections with an estimated 10% mortality rate [14]. Advanced age and comorbidities were significantly associated with increased risk of SARS-CoV related death, due to acute respiratory distress syndrome [15-18].

It is well appreciated that pulmonary damage in SARS-CoV infection is caused by direct viral effects as well as immunopathological factors [15], however the pathogenic mechanisms in the vulnerable aged populations remain poorly defined. Several aged animal models of SARS-CoV infection have been established to evaluate the response and elucidate mechanisms for increased SARS-CoV pathogenicity in the aged host. Recombinant infectious clones and mouse passaged isolates of SARS-CoV show increased severity of disease and lethality in aged as compared to young mice [19-21]. Interestingly, the aged and young hosts show similar levels of SARS-CoV replication in most experimental infections [22]. Thus the increased acute lung injury in SARS-CoV-infected aged animals is thought to be related to the over exuberant immune responses and not heightened viral-mediated damage. However, many aspects of the elderly immune response and how it may differ from the young adult are still unclear. Furthermore, our study represents one of only two SARS-CoV infection studies in aged nonhuman primates as almost all aged SARS-CoV experiments to date have been conducted in mouse models with mouse-adapted viral strains. Although murine models are often advantageous and informative, nonhuman primates may be better suited for studying the aging immune and respiratory systems. Unlike mice, nonhuman primates show a high level of genetic homology to humans, are not inbred, have longer life spans and their lungs are more structurally similar to humans than other laboratory animals [23]. Importantly, studies have shown that nonhuman primates undergo immune senescence similar to what has been described for humans [24,25]. The aim of this study was to determine how the peripheral and mucosal immune responses to SARS-CoV infection compare in the aged and juvenile nonhuman primate host and to determine how this may impact viral replication levels. We report that SARS-CoV virus titers were significantly higher in the nasal cavity of aged monkeys at day 1 post infection but, by day 3, the difference in titers between age groups was negligible. Although SARS-CoV virus levels were similar in aged and juvenile monkeys at later time points post infection there were significant age-dependent differences in systemic and mucosal immune responses to SARS-CoV.

## Results

### SARS-CoV infection and clinical features in juvenile and aged monkeys

**Figure 1:**
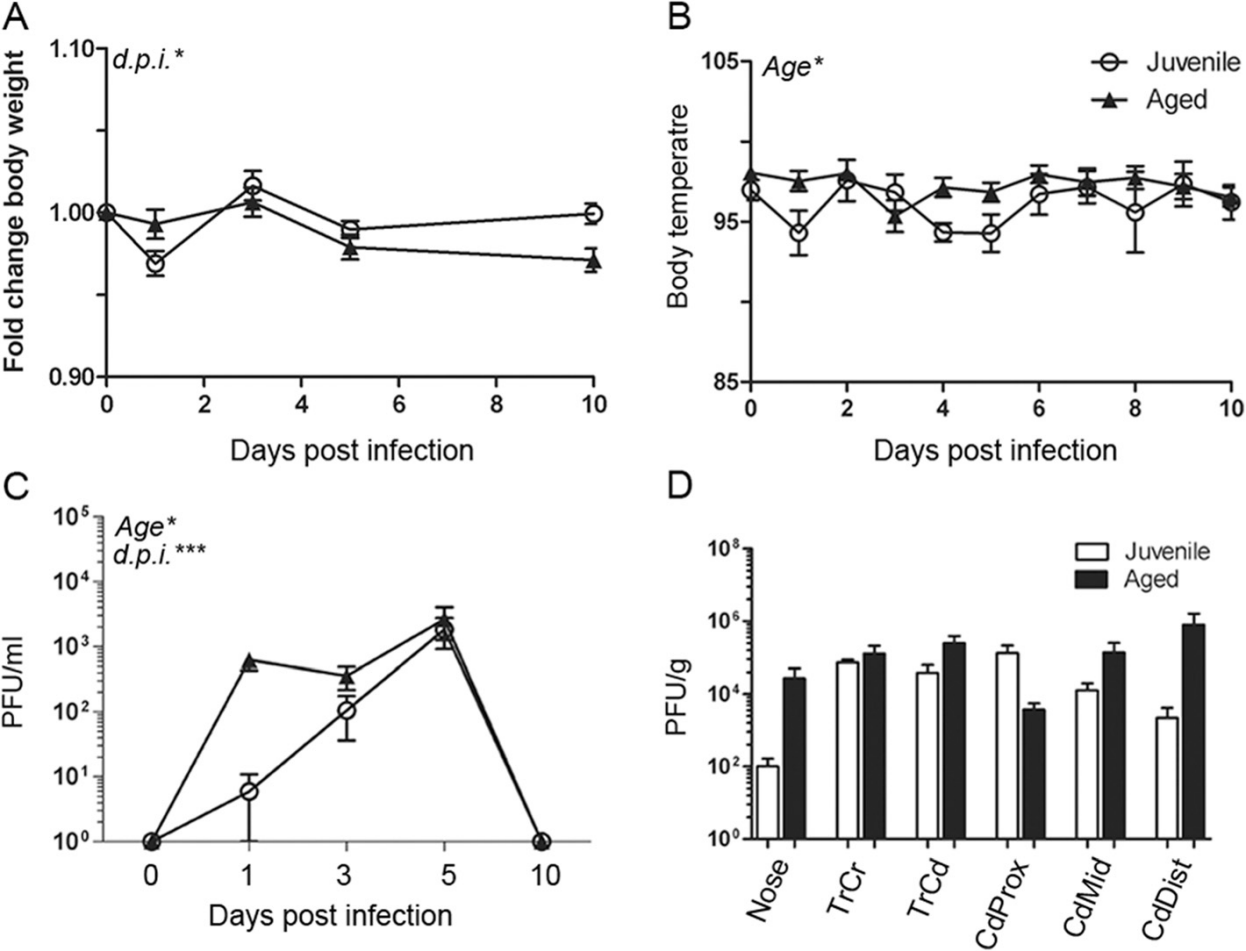
**Comparison of clinical features and replication levels following SARS-CoV-infection of aged and juvenile monkeys. (A)** Animals were weighed throughout the infection time course and mean values (+/− standard error (SE)) are graphed as the fold change over pre-infection body weight. **(B)** Body temperature was measured with an implanted subcutaneous temperature microchip transponder. Average daily values (+/− SE) are plotted in degrees Fahrenheit for aged and juvenile monkeys throughout SARS-CoV infection. **(C)** Viral titers were assessed longitudinally over the infection time course in nasal swabs by plaque-forming assays. The average plaque-forming units (PFU) per ml per nasal swab (+/−SE) are graphed on a log scale. Day 0–5 post infection for aged animals n = 10, day 10 n = 5. Day 0–5 post infection n = 12, for juvenile animals, day 10 n = 6. **(D)** SARS-CoV replication was also evaluated at 5 days post infection in several respiratory tract tissues including the nose, cranial trachea sample (TrCr), caudal trachea sample (TrCd), and proximal (CdProx), middle (CdMid) and distal (CdDist) portions of the right caudal lung lobe. All tissues were collected in a standardized manner. Bar graphs of the mean (+/−SE) values of plaque-forming units per gram tissue are plotted on a log scale. Aged n = 5, juvenile n = 6 for each tissue. *p < 0.05, **p < 0.01, ***p < 0.0005 by 2-way ANOVA for age or time post infection (d.p.i) as indicated in each graph.

To evaluate the impact of advanced age on severity of SARS-CoV infection, aged and juvenile African green monkeys were inoculated intranasally with 10^7^ plaque-forming units (PFU) SARS-CoV HKU-39849 strain or mock-infected with sacrifice at 5 or 10 days post infection (d.p.i.). Aged animals were roughly equivalent to 50 year old humans (n = 5 each time point post infection, n = 2 for mock-infected) [26,27] while juvenile animals represented 6–12 year olds (n = 6 for all juvenile groups). Some of the virology, pathology and immunology data for the juvenile animals have been previously reported [28] but are included here for age-related comparisons. In examination of clinical features, all animals showed a slight initial decrease in body weight with SARS-CoV inoculation that rebounded by 3 d.p.i. (Figure 1A; 2-way ANOVA for age and d.p.i.). Time post infection but not age was a significant source of variation for the fold change in body weight. Body temperature however, fluctuated throughout the infection time course and was significantly affected by age but not infection (Figure 1B; 2-way ANOVA for age and d.p.i.). SARS-CoV titers were measured in nasal swabs and in several respiratory tissues by plaque assays. Replicating virus was recovered from nasal swabs in 7 of the 10 aged animals at 1 d.p.i., in contrast to only one juvenile animal with detectable virus at this early time point (Figure 1C; 2-way ANOVA for age and d.p.i.). Day 1 was the only time point in which SARS-CoV replication was significantly different; by 3 d.p.i., virus levels were only slightly higher in aged animals and titers remained similar in both age groups out to day 5. No significant age-dependent differences in SARS-CoV levels were observed in standardized collected respiratory tissues at 5 d.p.i. (Figure 1D; 1-way ANOVA for age). However, the levels of virus tended to be higher in all tissues from the aged as compared to the juvenile animals, except in the proximal portion of the right caudal lung lobe. No virus was detected in any sample collected from either age group at 10 d.p.i.

### Comparison of lung lesions in the aged and juvenile host following SARS-CoV infection

To determine if advanced age correlated with increased severity of lung pathology, a comprehensive histological analysis of the respiratory tract following SARS-CoV infection was conducted in aged and juvenile animals. At 5 d.p.i. the SARS-CoV-induced histologic changes in the lung were higher in incidence in aged compared to juvenile monkeys (Table 1). These changes included perivascular cuffing with inflammatory cells, alveolitis and interstitial pneumonia. However, venous thrombosis was observed in 17-18% of the juvenile animals at both 5 and 10 d.p.i. whereas no venous thrombosis was detected in any aged animal at either time point. The severity of lung pathology was only slightly higher in aged as compared to juvenile animals at 5 d.p.i., and by 10 d.p.i., juvenile animals exhibited higher incidence and severity for most of the histologic changes. A marked increase in the incidence of interstitial pneumonia was observed in juvenile but not aged monkeys at 10 d.p.i. (83% in juvenile versus only 20% in aged). Taken together, the histologic changes associated with SARS-CoV-induced interstitial pneumonia were slightly lower in incidence and severity in the aged compared to juvenile host.

**Table 1:** Comparison of SARS-CoV induced histologic changes in the lungs of aged and juvenile monkeys

	**Value**					
**Group**	**No. of animals**	**Grade**^**a**^	**Venous thrombosis**	**Perivascular cuffing**	**Alveolitis**	**Interstitial pneumonia**
Aged 5 d.p.i.	5	Severity	0	1.8	1.2	0.4
		Incidence	0%	100%	80%	40%
Juvenile 5 d.p.i.	11	Severity	0.27	1.2	1	0.45
		Incidence	18%	82%	73%	27%
Aged 10 d.p.i.	5	Severity	0	1.6	1.4	0.2
		Incidence	0%	100%	80%	20%
Juvenile 10 d.p.i.	6	Severity	0.33	1.7	1.3	1.5
		Incidence	17%	100%	100%	83%
Aged mock	3	Severity	0	0.33	0	0
		Incidence	0%	33%	0%	0%
Juvenile mock	2	Severity	0	0	0	0
		Incidence	0%	0%	0%	0%

^a^Severity is the average grade for the group (0 = normal, 1 = minimal, 2 = mild, 3 = moderate, 4 = marked); incidence is the percentage of the group affected.

### Systemic and mucosal SARS-CoV-induced inflammatory responses differ in aged compared to juvenile monkeys

**Figure 2:**
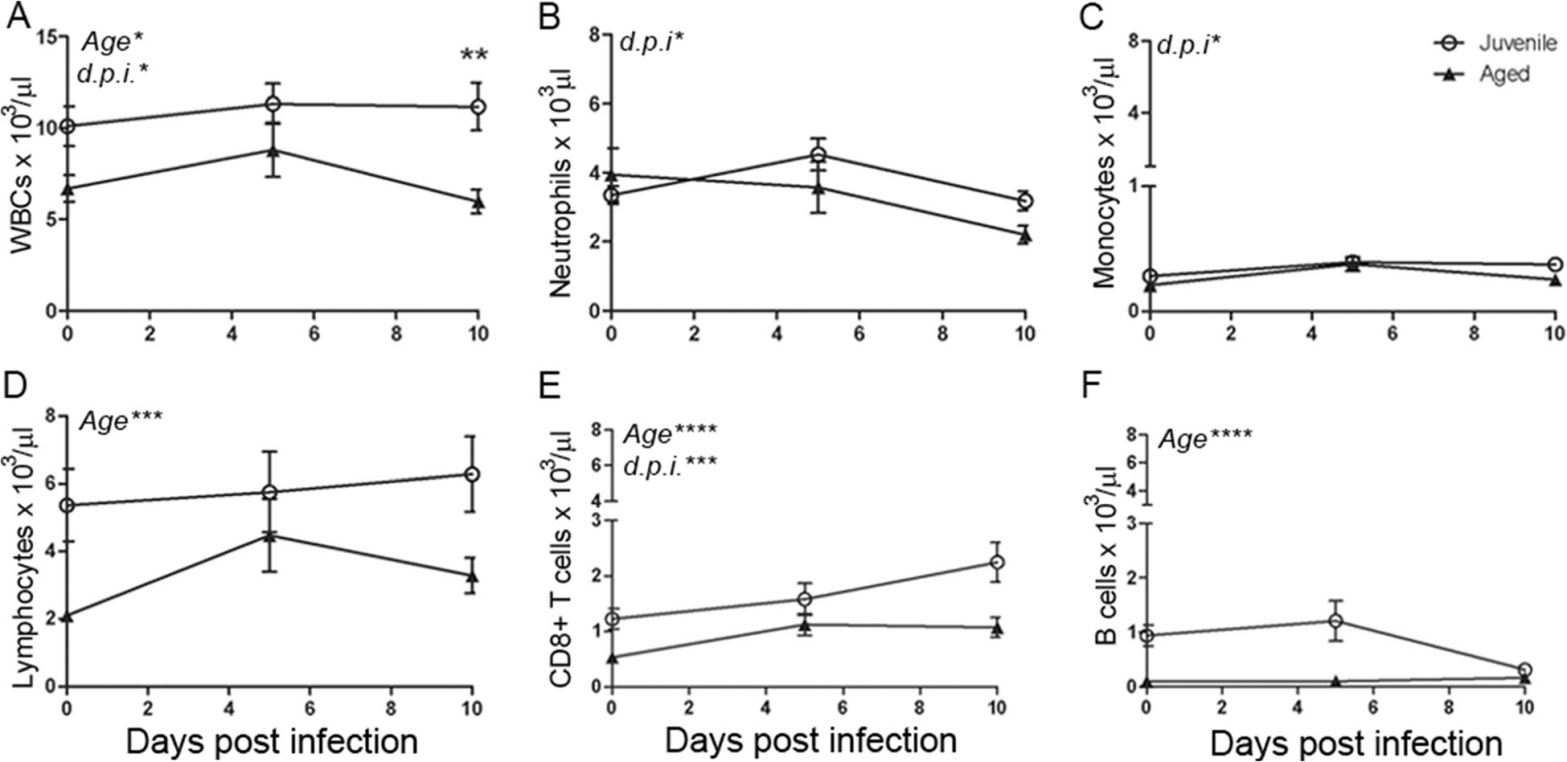
**Differences in peripheral blood cell populations in the aged and juvenile host following SARS-CoV infection.** Peripheral blood was collected prior to and at 5 or 10 days post SARS-CoV infection while animals were under sedation. Complete white blood cell counts and cell differentials were assessed by the Lovelace Clinical Laboratory Staff. The number of cells × 10^3 per microliter (μl) blood are graphed for **(A)** white blood cells (WBC), **(B)** neutrophils, **(C)** monocytes, and **(D)** lymphocytes. **(E-F)** The number of CD8 T cells and B cells per microliter blood was calculated based on flow cytometric frequencies and total lymphocyte numbers. The gating strategy is depicted in Additional file 1: Figure S1. Graphs represent average values (+/−SE). *p < 0.05, ***p < 0.0005, ****p < 0.0001 in 2-way ANOVA comparing age and time post infection (d.p.i.) as indicated in the graphs.

To determine if there were age-specific differences in immune activation following SARS-CoV infection, systemic inflammatory responses were compared in aged and juvenile animals. Peripheral blood draws prior to and at days 1, 5 and 10 were used to monitor circulating immune cell and cytokine profiles. The total white blood cell counts for aged monkeys were lower than their juvenile counterparts at pre- and post SARS-CoV infection time points (Figure 2A; 2-way ANOVA for age and d.p.i.). In examination of specific leukocyte populations, neutrophil and monocyte numbers significantly varied with SARS-CoV infection but were unaffected by age (Figure 2B-C; 2-way ANOVA for age and d.p.i.). In contrast, age was a significant source of variation for lymphocyte numbers with reduced CD8 T cells and B cells in the peripheral blood of aged compared to juvenile animals (Figure 2D-F; 2-way ANOVA for age and d.p.i.).

The serum cytokine profile during SARS-CoV infection in aged and juvenile monkeys was defined using bead-based protein arrays focusing on inflammatory cytokines and chemokines associated with antiviral responses (Table 2). In comparing the aged and juvenile animals, the levels of IL-12 and IFN-gamma were similar but had a trend of being higher in the aged monkeys whereas CCL2 was higher in juveniles, although not reaching statistical significance (2-way ANOVA for age and d.p.i.). IL-1 receptor agonist at baseline and CCL3 levels at day 10 were significantly elevated in the serum of aged animals over their juvenile counterparts (2-way ANOVA for age and d.p.i. with Bonferroni post-tests). As expected with an experiment using non-inbred animals the variation for serum cytokines was relatively high, particularly for the aged animals which is consistent with other previously published aged nonhuman primate studies [5,25].

**Table 2:** Cytokines and chemokines in serum of SARS-CoV infected aged and juvenile monkeys

	**Concentration pg**/**ml cytokine +/− SE**						
**Cytokine or chemokine**	**Day 1**		**Day 5**		**Day 10**		***Statistics***
	**Juvenile**	**Aged**	**Juvenile**	**Aged**	**Juvenile**	**Aged**	
IL-1 ra	1.7 ± 1.3	**12.0 + 3.1**	59 ± 3.6	16.9 + 3.7	7.1 ± 3.9	6.7 ± 2.1	*age p = 0.06*
IL-8	156.0 ± 66.6	198.5 ± 81.4	200.2 ± 180.8	204.6 ± 98.5	151.1 ± 130.2	105.1 ± 73.7	
IL-12	34.1 ± 6.8	66.4 ± 15.0	37.5 ± 14.6	45.0 ± 21.2	24.4 ± 8.8	75.9 ± 42.1	
IL-15	2.2 ± 0.3	1.4 ± 0.3	3.9 ± 0.7	3.8 ± 1.2	4.0 ± 1.3	3.2 ± 0.4	d.p.i.^*^
IL-23	34.1 ± 6.8	58.1 ± 15.4	37.5 ± 14.6	45.0 ± 21.2	24.4 ± 8.8	75.9 ± 42.1	
IFN-y	0.7 ± 0.3	1.1 ± 0.3	1.6 ± 0.9	2.7 ± 0.8	2.5 ± 1.1	3.8 ± 2.6	
CCL2	1657.5 ± 129.5	1631.0 ± 463.9	3279.1 ± 619.9	1920.4 ± 535.5	1826.0 ± 5972	1160.0 ± 415.0	d.p.i.^*^
CCL3	2.6 ± 0.7	9.4 ± 2.3	2.1 ± 1.2	4.9 ± 2.9	0.5 ± 0.2	6.6 ± 2.1*	age^*^, d.p.i.^*^

Values represent picograms +/- standard error. Results of a 2-way ANOVA for age and days post infection (d.p.i.) are indicated at the right with asterisks denoting degree of significance p < 0.05. Significantly higher levels of cytokine at specific time points as determined by Bonferonni post tests are in bold.

**Figure 3:**
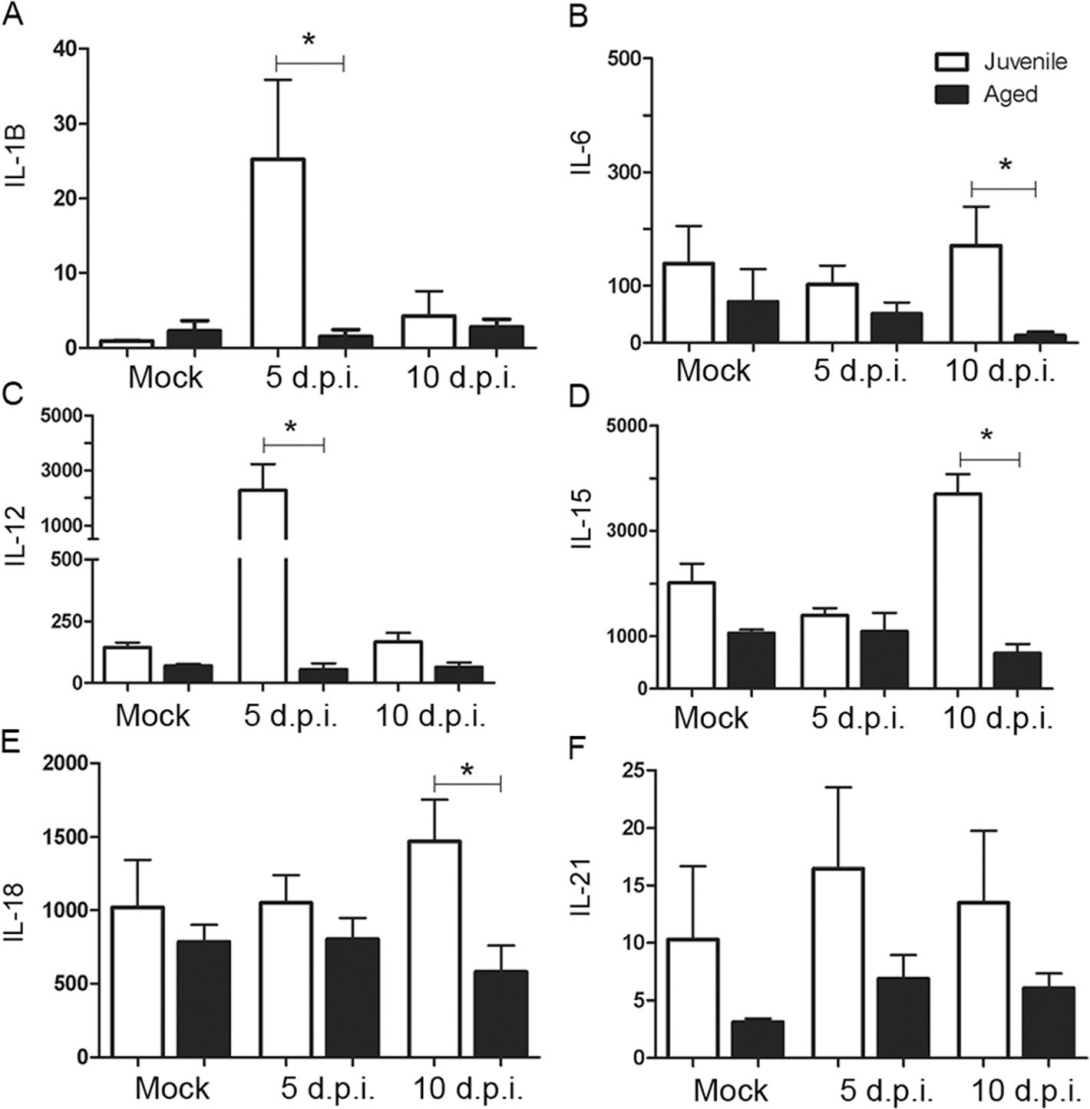
**SARS-CoV induced proinflammatory and antiviral cytokine expression differs in the lungs of aged compared to juvenile monkeys.** Cytokines were measured in standardized-collected lung tissue homogenates from the right caudal lung lobe of both age groups by bead-based arrays in mock and SARS-CoV infected animals at 5 and 10 days post infection (d.p.i.). Levels of proinflammatory cytokines IL-1Beta **(A)** and IL-6 **(B)** as well as antiviral cytokines IL-12 **(C)**, IL-15 **(D)**, IL-18 **(E)**, and IL-21 **(F)** were compared in aged and juvenile monkeys. Graphs represent average values (+/−SE) of cytokine in picograms per milliliter. *p < 0.05 in unpaired student *T*-test comparing juvenile and aged animals at the time points indicated by the horizontal bar.

To determine how mucosal cytokines in SARS-CoV infection compared to systemic responses and how age may impact mucosal cytokine expression; the inflammatory protein profile was evaluated by bead-based arrays in standardized-collected lung tissue from the proximal portion of the right caudal lobe. The focus was on inflammatory cytokines thought to play a role in antiviral immunity. In contrast to the higher cytokine trend in the serum of aged animals, cytokines in the lung were often lower in aged as compared to the juvenile monkeys. Proinflammatory cytokines IL-1beta and IL-6 were significantly lower in aged as compared to juvenile animals at 5 and 10 d.p.i. respectively (Figure 3A-B; unpaired student *T*-test). Aged monkeys showed significantly lower levels of IL-12 at day 5 and lower IL-15 and IL-18 at 10 d.p.i. as compared to juveniles (Figures 3C-E; unpaired student *T*-test). There was also a trend of decreased IL-21 levels in the lungs of aged versus juvenile monkeys although not reaching statistical significance (Figures 3F). Unlike the juvenile animals, interferon-gamma was below the level of detection in aged lung samples (data not shown). In general, cytokines tended to increase with SARS-CoV infection in juvenile monkeys whereas levels in the aged group showed no infection-induced increase.

### Age-specific differences in lung chemokine profiles during SARS-CoV infection

**Figure 4:**
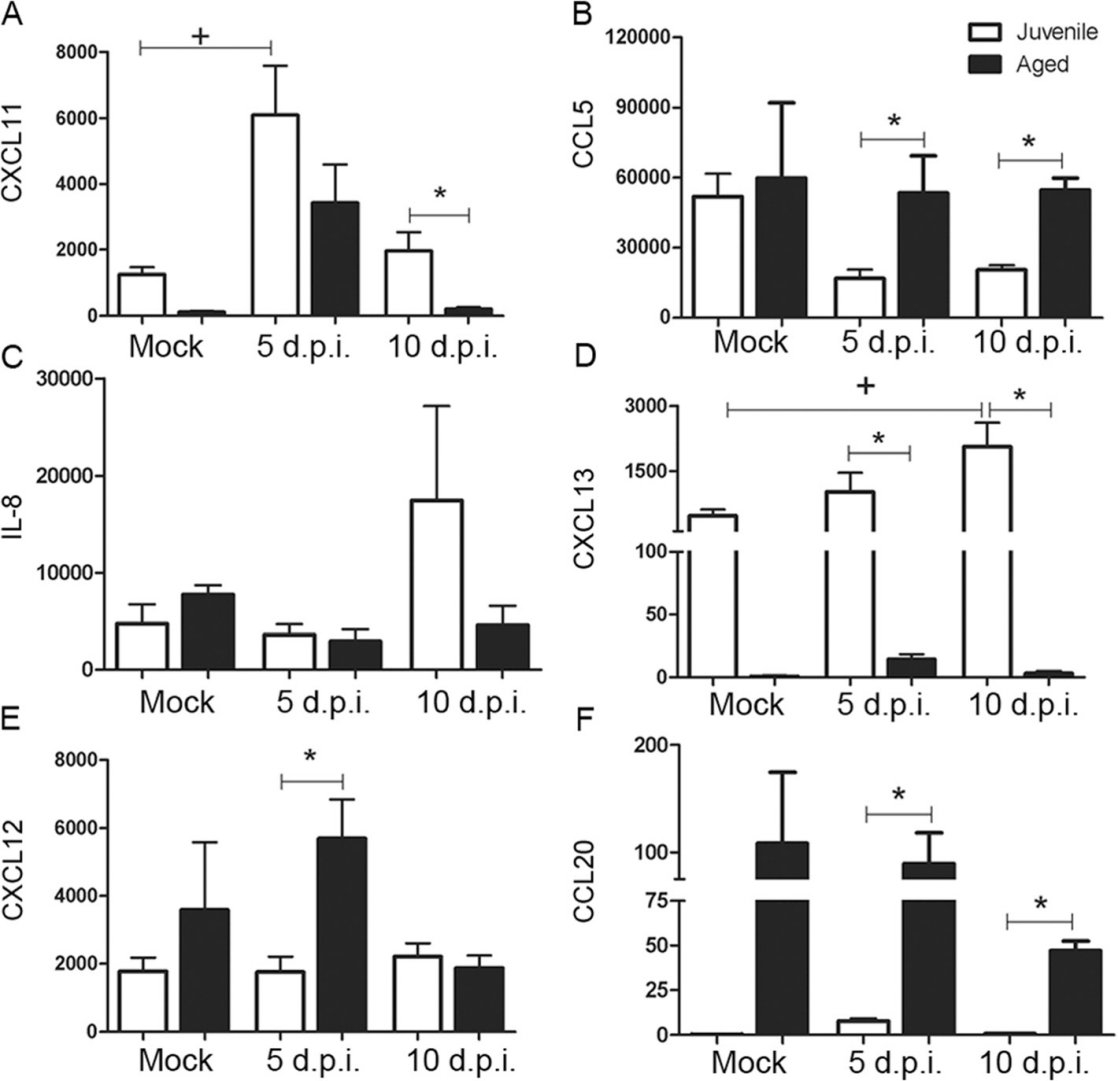
**Aged and juvenile monkeys show distinct lung chemokine profiles during SARS-CoV infection.** Chemokines CXCL11 **(A)**, CCL5 **(B)**, IL-8 **(C)**, CXCL13 **(D)**, CXCL12 **(E)**, CCL20 **(F)** were measured in standardized collected lung tissue homogenates of the right caudal lung lobe by bead-based arrays as in Figure 3. Graphs represent average values of chemokine in picograms per milliliter (+/−SE). *p < 0.05 for comparison of age and + *p* < 0.05 for comparion of time post infection in unpaired student *T*-test.

Chemokines play a critical role in coordinating the migration of leukocytes into and out of the lung to trigger effective immune responses against viral pathogens [29,30]. The chemokine milieu in the lungs following SARS-CoV infection was evaluated in aged and juvenile animals by targeted protein arrays. Unlike data for proinflammatory cytokines in the lung, several of the chemokines showed increased expression with SARS-CoV infection and were detected at higher levels in the aged compared to the juvenile host. The T cell chemoattractant, CXCL11 was elevated in both age groups with SARS-CoV infection (Figure 4A). However, CXCL11 levels in the aged lung did not reach the same magnitude as the juveniles and were significantly lower in comparison at 10 d.p.i. (unpaired student *T*-test). In contrast, CCL5, another activated T cell chemoattractant was significantly higher in aged compared to juvenile animals throughout the SARS-CoV infection period (Figure 4B; unpaired student *T*-test). The B cell chemoattractant, CXCL13 was dramatically lower in aged as compared to juvenile animals, regardless of infection status whereas CXCL12 and CCL20, known dendritic cell (DC) chemoattractants, were much higher in mock and SARS-CoV infected aged animals (Figure 4D-F; unpaired student *T*-test).

### Inflammatory lung infiltrates in SARS-CoV infection differ in aged compared to juvenile monkeys

**Figure 5:**
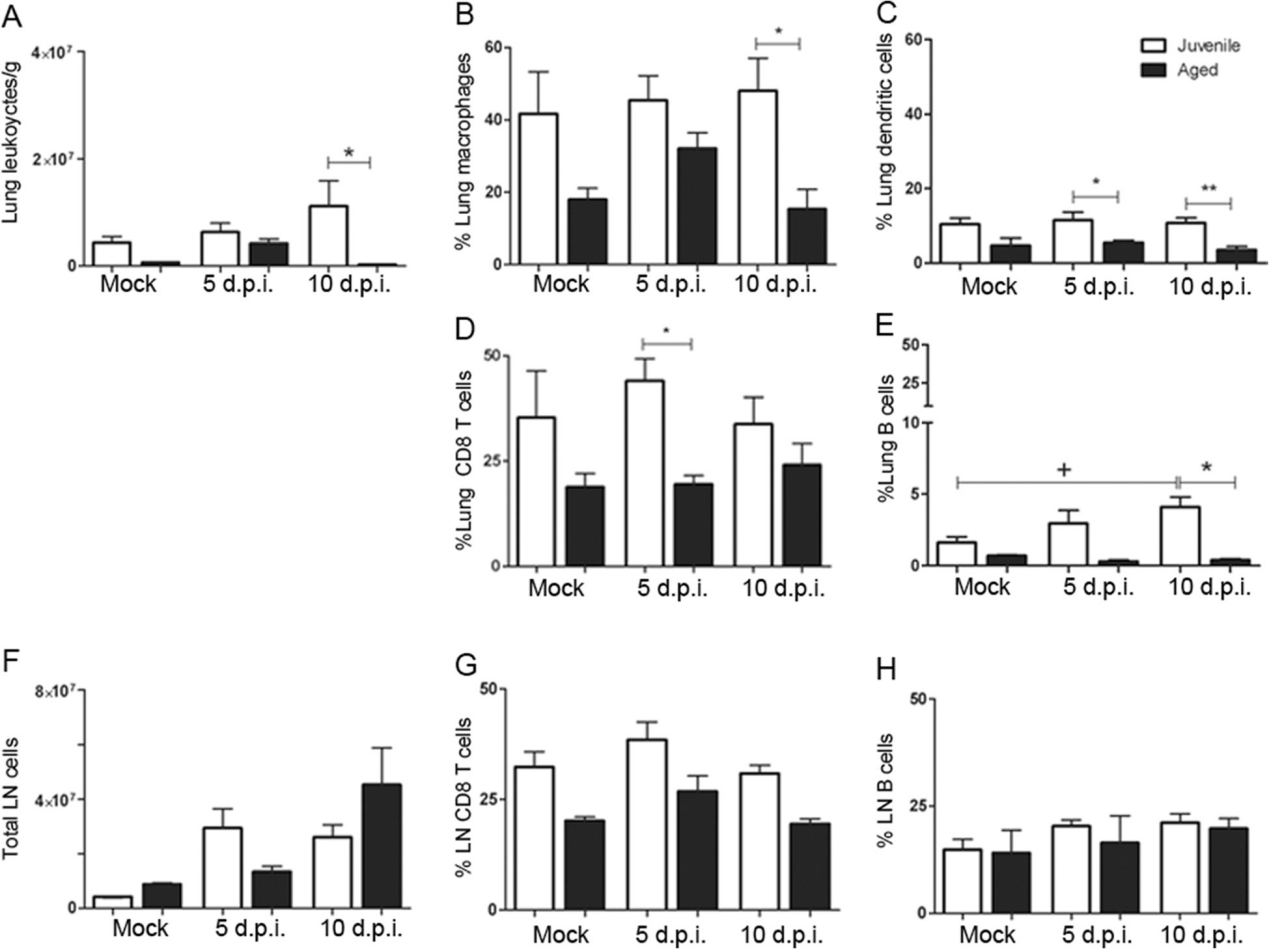
**Age-specific differences in leukocyte populations in the lung but not lymph node following SARS-CoV infection. (A)** The number of total lung leukocytes was calculated per gram of tissue in standardized collected samples from mock- and SARS-CoV infected animals at 5 and 10 days post infection (d.p.i.). Samples were taken from the proximal portion of the right caudal lobe adjacent to the region used for cytokine analysis (Figures 3–4). Flow cytometry was used to further characterize the major leukocyte populations in the lung mucosa. The percent frequency of total lung leukocytes are indicated for **(B)** macrophages (CD68 + HLADR+), **(C)** dendritic cells (DCs; CD68-HLADR + CD11c+), **(D)** CD8 T lymphocytes (CD3 + CD8+) and **(E)** B cells (CD3-CD20+). **(F)** The number of total cells collected from the tracheobronchial lymph node (LN) tissue was quantitated for mock- and SARS-CoV infected animals at 5 and 10 d.p.i.. The percent frequency of total lymph node cells were determined for **(G)** CD8+ T cells and **(H)** B cells. Graphs represent average values (+/−SE). The gating strategies for flow cytometric analysis are shown in Additional file 1: Figures S1 and Additional file 2: Figure S2 and percent frequencies summarized in Additional file 3: Table S1. *p < 0.05, **p < 0.01 in unpaired student *T*-test comparing age groups. +*p* < 0.05 for comparing infection time points.

To determine if age impacted the kinetics and magnitude of mucosal inflammation in response to SARS-CoV, immune cell populations were quantified and characterized by flow cytometry in standardized collected lung tissue (proximal portion of the right caudal lobe) and lung draining lymph nodes. The number of total lung leukocytes per gram lung tissue was similar at 5 d.p.i. but was significantly lower in the aged as compared to juvenile animals at 10 d.p.i. (Figure 5A; unpaired student *T*-test). The frequency of distinct lung leukocyte populations, including macrophages (CD68 + HLADR+), dendritic cells (DCs; CD68-HLADR + CD11c+), CD8 T lymphocytes (CD3 + CD8+), and B cells (CD3-CD20+) differed in the two age groups during SARS-CoV infection (Figure 5B-E). Gating strategies are shown in Additional file 1: Figures S1 and Additional file 2: Figure S2 and average values are summarized in Additional file 3: Table S1. The frequency of lung macrophages was lower in aged as compared to juvenile animals in mock and SARS-CoV infection, reaching significance at 10 d.p.i. (Figure 5B; unpaired student *T*-test). The proportion of lung DCs was also significantly lower in aged animals at all infection time points examined (Figure 5C; unpaired student *T*-test). Although lung B cell frequencies were increased with infection in juvenile animals, no increase was observed in aged monkeys and, at most infection time points, significantly lower CD8 T cell and B cell frequencies were detected in aged monkeys (Figure 5D-E; unpaired student *T*-test). In contrast to the lung, no age-dependent differences in the leukocyte numbers or frequency of T and B cells were noted in the tracheobronchial lymph nodes (Figure 5F-H; unpaired student *T*-test).

To further assess the age-specific differences in immunity to SARS-CoV infection, the activation status and chemokine receptor profile was assessed on monocytes/macrophages, DCs and CD8 T cells. Peripheral monocytes were defined by flow cytometry using CD14 and HLADR expression and DCs as CD14-HLADR + CD11c + (see peripheral blood monocyte and DC gating strategy in Additional file 4: Figure S3). Expression of the activation marker CD86 showed significant age-dependent differences in the monocytes, lung macrophages and DC populations with significantly less expression in aged SARS-CoV-infected animals (Table 3; 2-way ANOVA; Additional file 2: Figures S2 and Additional file 4: Figure S3). We also examined peripheral monocytes, DCs and CD8 T cells for CCL5 and CCL20 receptor expression as these chemokines were found at highest concentrations in the aged lung. For CCL5 receptors, aged monocytes showed reduced CCR3 but similar levels of CCR1 compared to their juvenile counterparts (Table 3, 2-way ANOVA for age and d.p.i.). The frequency of CD8 T cells expressing CCR5 (another CCL5 receptor) was not significantly impacted by age. The receptor for CCL20, CCR6 was significantly down regulated with age and SARS-CoV infection on peripheral blood DCs (Table 3, 2-way ANOVA for age and d.p.i.).

**Table 3:** Percent frequency of chemokine receptor and activation marker positive leukocytes in SARS-CoV infected aged and juvenile monkeys

	**Frequency of cells**^**a**^						
**Peripheral blood**	**Day 1**		**Day 5**		**Day 10**		
	**Juvenile**	**Aged**	**Juvenile**	**Aged**	**Juvenile**	**Aged**	**Statistics**
Monocytes							
CCR1	5.0 ± 0.9	1.8 ± 0.4	1.0 ± 0.1	2.2 ± 1.7	*2.5* ± *0.3*	2.4 ± 0.6	d.p.i*
CCR3	1.6 ± 0.4	1.9 ± 04	1.1 ± 01	2.2 ± 12	1.1 ± 01	2.4 ± 0.6	age*
CD86	8.9 ± 13	3.8 ± 0.4	6.3 ± 2.0	3.9 ± 1.9	4.7 ± 0.3	5.2 ± 1.6	age*
DCs							
CCR6	0.6 ± 0.3	0.3 ± 0.2	0.6 ± 0.2	0.0 ± 0.0	0.3 ± 01	0.2 ± 0.0	age**, d.p.i^*^
CD86	1.4 ± 0.5	0.2 ± 0.0	2.9 ± 0.9	0.3 ± 0.0	0.2 ± 0.0	0.2 ± 0.0	age****
CD8 + T cells							
CCR5	6.9 ± 1.2	2.5 ± 0.4	1.8 ± 0.3	2.0 ± 0.5	3.7 ± 0.3	9.9 ± 3.4	d.p.i**
**Lung**	Mock		Day 5		Day 10		
	Juvenile	Aged	Juvenile	Aged	Juvenile	Aged	Statistics
Macrophages							
CD86	20.5 ± 1.7	2.3 ± 0.1	36.4 ± 4.8	3.1 ± 1.1	34.2 ± 2.1	3.3 ± 1.7	age^****^
DCs							
CD86	1.1 ± 0.3	0.3 ± 0.1	3.7 ± 0.5	0.4 ± 0.1	2.0 ± 0.5	0.3 ± 0.1	age^****^

^a^Average frequency of chemokine receptor or activation marker positive cells of total perhipheral blood or lung leukocytes. Monocytes (CD14 + HLADR+), peripheral blood dendritic cells (DCs: CD14-HLADR + CD11c+), CD8 T cells (CD3 + CD8+). Lung macrophages are defined as CD68 + HLADR + and lung DCs as CD68-HLADR + CD11c+. Gating strategies are show in Additional file 1: Figure S1, Additional file 2: Figure S2 and Additional file 4: Figure S3. Statistical results of 2-way ANOVas for age and days post infection (d.p.i.) are indicated at the right with asterisks denoting degree of sigrificance: *p < 0.05, **p < 0.005 ****p < 0.0001.

### Impact of age on SARS-CoV specific T and B cell responses

**Figure 6:**
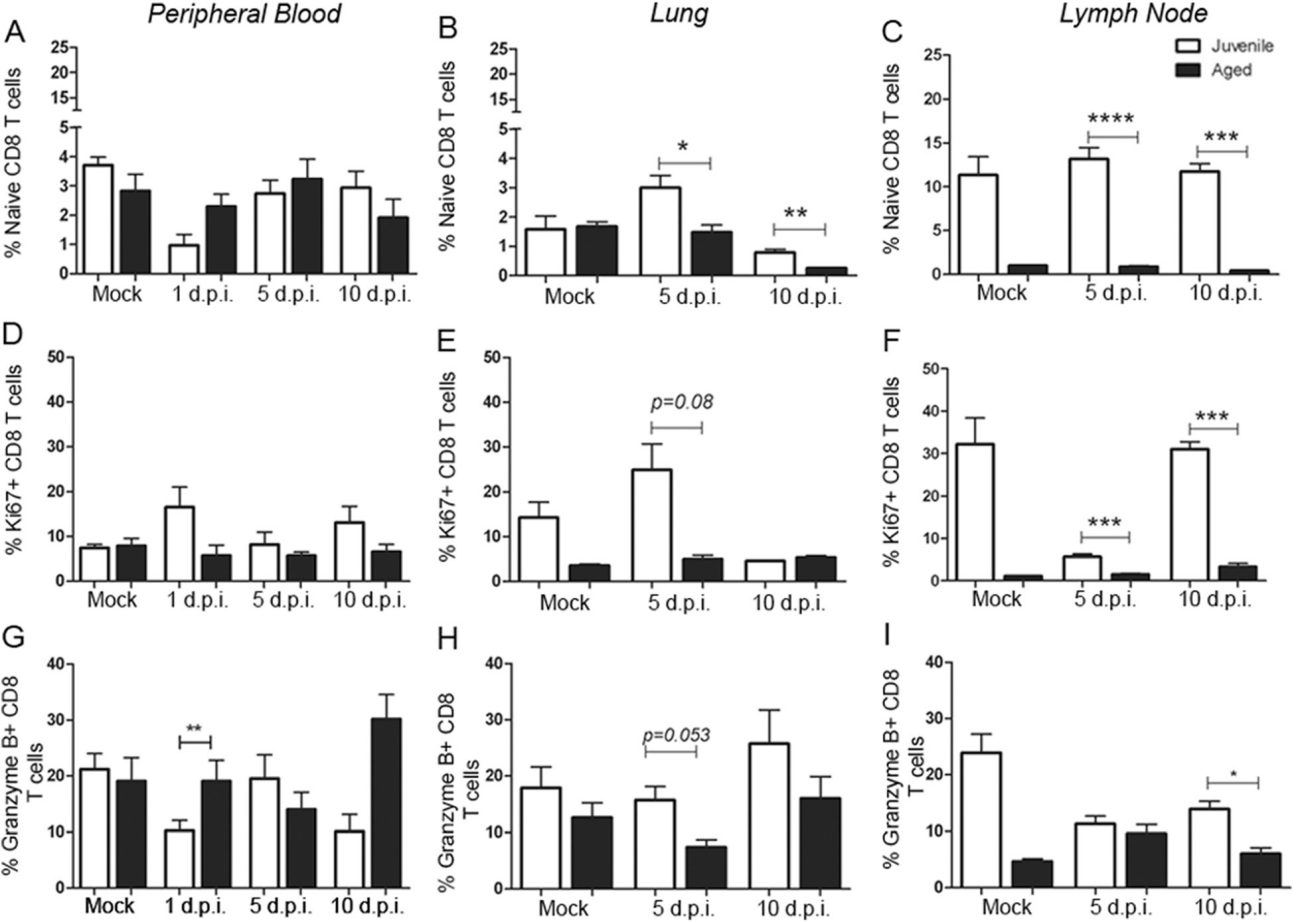
**Comparison of T lymphocyte responses to SARS-CoV infection in the aged and juvenile monkey.** CD8 T cells were evaluated by flow cytometric analysis for coexpression of naïve cell markers CD45RA and CCR7 **(A-C)**; proliferation marker, Ki67 **(D-F)**; and cytotoxic enzyme, granzyme B **(G-I)** in the peripheral blood, lung and tracheobronchial lymph nodes. Graphs represent average values (+/−SE). The gating strategy for flow cytometric analysis is shown in Additional file 1: Figures S1 and Additional file 2: Figure S2. *p < 0.05, **p < 0.01, ***p < 0.001, ****p < 0.0001 in unpaired student T-tests comparing age groups.

**Figure 7:**
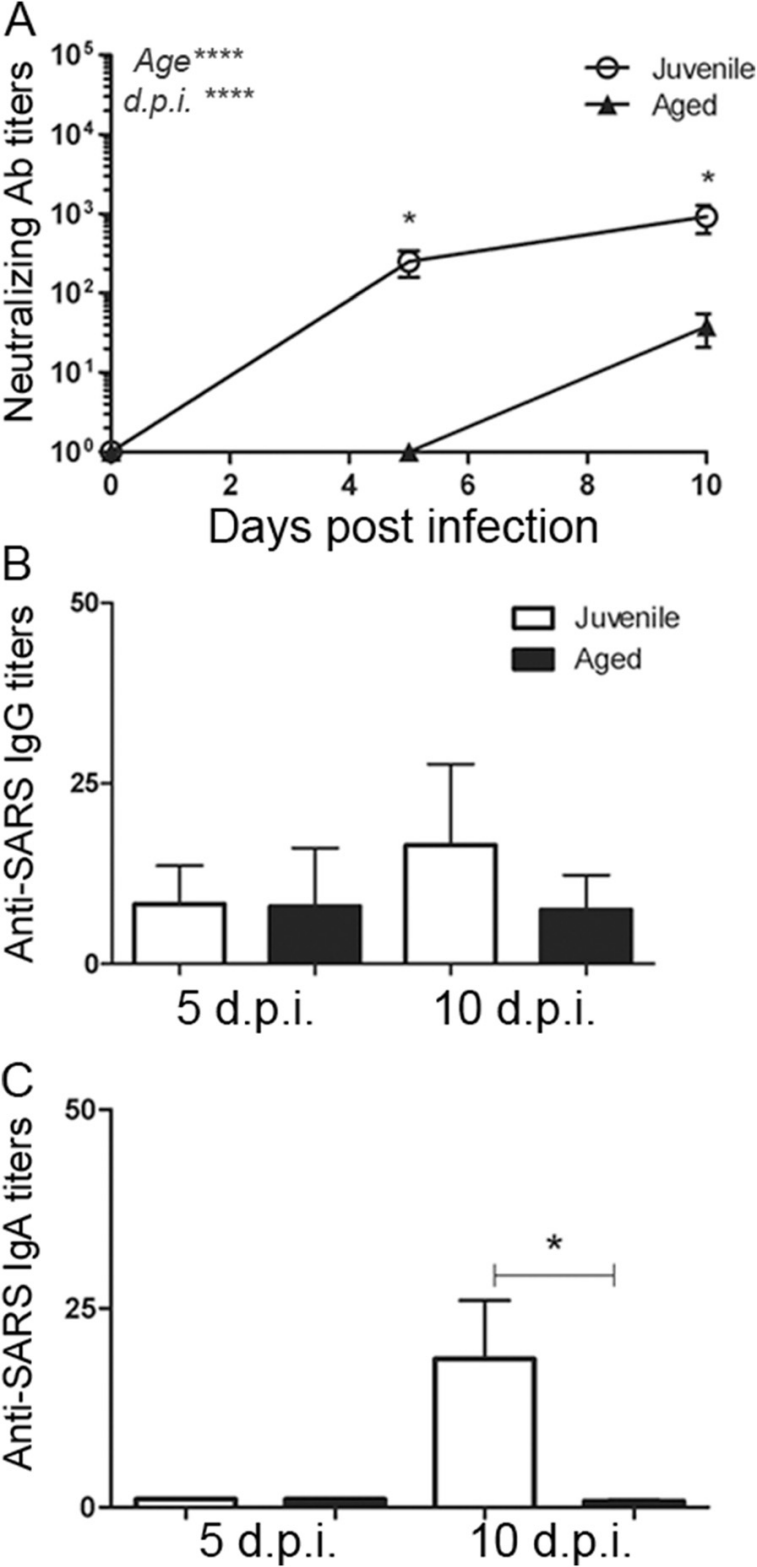
**Limited systemic and mucosal B cell responses in aged monkeys infected with SARS-CoV. (A)** SARS-CoV neutralizing antibodies were measured in sera of aged and juvenile animals over the infection time course. Graphs represent average values (+/−SE). For day 0, aged n = 10, juvenile n = 12. For day 5 and 10 post infection n = 5 for aged and n = 6 for juvenile. ****p < 0.0001 for age and time post infection (d.p.i.) as indicated in the graph by 2-way ANOVA with a Bonferroni post-test, p < 0.05. **(B-C)** Anti-SARS-CoV spike protein specific IgG **(B)** and IgA **(C)** were measured in lung tissue homogenates by ELISA. Graphs represent average values, n = 5 for aged and n = 6 for juvenile (+/−SE). *p < 0.05 in unpaired student *T*-test comparing juvenile and aged animals at the time points indicated by the horizontal bar.

CD8 T cell responses to SARS-CoV infection were evaluated in peripheral blood, lung and tracheobronchial lymph node by flow cytometric analysis (See gating strategy in Additional file 1: Figures S1 and Additional file 2: Figure S2). Although no age-dependent differences were observed in the frequency of naïve (CD45RA + CCR7+) CD8 T cells in peripheral blood, there were significantly lower levels of these cells in the lung and lymph node of aged animals during SARS-CoV infection (Figure 6A-C; unpaired student T-tests). A similar trend was observed with proliferating and cytotoxic granzyme B + CD8 T cells which were lower in the lungs and lymph nodes of aged compared to their juvenile counterparts at most infection time points (Figure 6D-I; unpaired student *T*-test). However, granzyme B + T cell levels tended to be higher in the peripheral blood of aged compared to juvenile animals, reaching significance at 1 d.p.i. (Figure 6G; unpaired student *T*-test). To determine how the B cell response to SARS-CoV was impacted by age, anti-SARS-CoV antibodies were evaluated in the serum and lung. Serum neutralization assays revealed increased antibodies in all juvenile animals with SARS-CoV infection as early as 5 d.p.i. (Figure 7A; 2-way ANOVA for age and d.p.i.). In contrast, SARS-CoV neutralizing antibodies were not detected until 10 d.p.i. in aged monkeys, with antibody titers that were significantly lower as compared to juveniles. Anti-SARS-CoV IgG and IgA antibodies in lung tissue homogenates were determined by ELISA. Lung anti-SARS-CoV IgG was detected in 1 aged and 2 juvenile animals at 5 d.p.i. and although titers increased in the juvenile lungs by day 10, no increase in anti-SARS CoV IgG titers was observed in aged animals (Figure 7B, no significance by unpaired student T test). Anti-SARS-CoV IgA titers were significantly lower in aged as compared to juvenile animals at 10 d.p.i., with average titers that were up to 18-fold lower in the aged host (Figure 7C, unpaired student T test).

## Discussion

The elderly (those over 65), are considered the fastest-growing demographic in the United States and are expected to make up 19% of the population by 2030 [31]. It is well recognized that elderly individuals incur enhanced severity of respiratory infections and according to the Center for Disease Control and Prevention, an estimated 9 out of 10 flu-related deaths in the United States occur in people 65 and older. When SARS-CoV emerged in the human population in 2002, the elderly were also disproportionately affected, with individuals over 65 making up 50% of the total fatal cases [32,33]. We have a poor understanding of the relationship between aging and the host response to respiratory virus infection. Most of our understanding of the biological changes that occur with aging in humans has been limited to studies of peripheral blood, which may not reflect the immune dynamics of the respiratory tract. Our study aimed at gaining insight into the kinetics and magnitude of the systemic and mucosal immune response to SARS-CoV infection in aged nonhuman primates, an important translational model for immune-aging research.

The significantly higher levels of viral replication in the nasal secretions of aged monkeys as compared to the juvenile animals at 1 d.p.i. was unexpected as most aged SARS-CoV studies have detected no age-dependent differences in infection levels. Higher SARS-CoV titers may have been missed in the previous aged nonhuman primate experiment as 2 d.p.i. was their earliest sampling time point, [22] and by day 3 in our study, aged and juvenile infection levels were similar. Although we cannot be certain that we were measuring replicating virus and not virus delivered from the inoculum, the dramatic difference in the amount recovered suggests that the aged nasal epithelium may support higher levels of SARS-CoV, or that the nasal cavity drainage mechanisms are impaired in the aged host. Despite the early age-associated differences in viral shedding at mucosal sites, by 5 d.p.i., levels of SARS-CoV in respiratory tract tissues were only slightly higher in the aged monkeys. Similar to previous reports, virus was not recovered from any animal at 10 d.p.i., suggesting that the kinetics of viral clearance may have been similar in both age groups. However, additional sampling between days 5 and 10 post infection would be necessary to confirm this observation.

The aged monkeys in our study exhibited significantly decreased total white blood cell counts which is consistent with the inverse correlation of WBCs with age that has been observed at steady state in several rhesus and human studies [25,34]. Of the leukocyte populations in the blood, lymphocyte numbers were most dramatically affected by age, with flow cytometric results showing significantly reduced CD8 T cells and B cells in aged compared to juvenile monkeys. In contrast, peripheral cytokine responses showed only minimal age-related differences to SARS-CoV infection which may be related to the high variability, particularly in the aged group, which is consistent with previous reports in elderly rhesus monkeys [5,25]. In regards to the mucosal inflammatory reactions, proinflammatory cytokines IL-1beta, IL-6, IL-15, IL-12 and IL-18 were all significantly lower in the lungs of SARS-CoV-infected aged animals at either day 5 or 10 post infection. In particular, the reduced levels of IL-1beta and IL-18 are in line with recent reports of impaired NLRP3 inflammasome function in elderly mice during influenza infection [35]. The low cytokine response in aged monkeys may also be a reflection of the reduced total inflammatory cell numbers found in the lungs of aged as compared to juvenile animals as cells and cytokines were evaluated in adjacent lung regions. Our data is not consistent with the exacerbated acute inflammatory responses shown to promote disease pathogenesis in aged SARS-CoV-infected mouse models [19,21]. As we did not sample earlier than day 5 post infection in the lung, the increased inflammation in our aged monkeys may have been missed. Our sampling schedule may have also precluded collections at optimal inflammatory response time points, as a biphasic pattern occurring in waves, at day 2 and 7 post infection with mouse-adapted SARS-CoV strains has been described in aged rodents [20,36].

In addition to the differences between our findings and those from aged mice, most of our results are also dissimilar to the previously reported aged nonhuman primate SARS-CoV study by Smits et al., [22] as we did not observe stronger innate immune responses or more severe pathology in our aged monkeys. In comparing our results, there are several experimental design aspects to take into consideration, beyond differences in dose and route of SARS-CoV infection. First, cynomolgous macaques were used in the previous study whereas our SARS-CoV infection model utilizes African green monkeys. In addition, timing of lung sample collection differed between the two studies, days 2 and 4 were assessed in the cynomolgous macaques while days 5, and 10 post infection were evaluated in our study. Furthermore, a standardized lung collection scheme was used in our immunology and pathology analysis, to give a more comprehensive perspective whereas their study assessed gene changes in specific regions of high SARS-CoV replication. Despite the major differences in study design, there were several results that were comparable, including the similarity of SARS-CoV viral titers in the aged and juvenile lung at later infection time points (days 4 and 5). In addition, several chemokines, including CCL5, CXCL12 and CCL20 were significantly increased in the lungs of SARS-CoV-infected aged monkeys here, which is consistent with the finding of elevated chemokine signaling genes in the aged lungs of cynomolgous macaques during SARS-CoV infection by microarray [22].

Immune senescence has been shown to impact innate as well as adaptive branches of the immune system, both contributing to the diminished immunity observed in the elderly [24,37]. In examination of the kinetics of lung and lymph node cell expansion and/or trafficking following SARS-CoV infection, we found significantly reduced lung macrophages, DCs, CD8 T cells and B cells in the aged animals relative to their juvenile counterparts. Not only were there fewer lung macrophages and DCs in the aged animals, but the frequency of costimulatory CD86+ cells were also significantly reduced. In contrast, the lymph node reaction showed no age-specific differences. Interestingly, although lung leukocyte numbers were reduced in aged monkeys there were disproportionately high levels of the chemokines capable of recruiting these cells into the lung, CCL5, CXCL11, CXCL12 and CCL20. This prompted our examination of the expression of the corresponding ligand-binding receptors on peripheral blood leukocytes to determine if there were age-related differences in the migratory capacity of these cells into the lung. For CCL5 receptors, we found significantly reduced frequencies of CCR3 but not CCR1+ monocytes in aged animals and similar frequencies of CCR5+ CD8 T cells in both age groups. The frequency of aged DCs expressing the CCL20 receptor, CCR6 showed the most dramatic age-specific reduction which is reasonable, given that CCR6 is predominantly expressed by immature DCs and may reflect a reduction of these cells in our aged animals. The observed differences in lung chemokine and ligand receptor expression may reflect age-specific modulation of leukocyte trafficking into the lung. Although we did not confirm these observations with mechanistic in vitro experiments, previous studies have shown that DCs acquired from older individuals display significantly impaired ability to migrate in response to chemokines [38].

We also examined age-specific differences in both T and B cell responses to SARS-CoV infection systemically and at mucosal sites. Not surprisingly, the proportion of naïve CD8 T cells in the lung and lymph node was significantly reduced in aged compared to juvenile animals in SARS-CoV infection. This corresponded to significantly lower levels of CD8 T cell proliferation at these sites in aged animals as well. Interestingly, the frequency of cytotoxic enzyme positive (granzyme B) T cells was higher at 1 d.p.i. in the periphery of aged compared to juvenile animals. However, aged peripheral granzyme + T cell frequencies remained unchanged by SARS-CoV infection whereas juvenile cytotoxic T cells showed infection-induced fluctuation. In contrast to peripheral blood, the lung and lymph node of aged animals had lower frequencies of granzyme + T cells compared to juvenile animals at 5 and 10 d.p.i.. The humoral response was also greatly reduced in aged SARS-CoV-infected monkeys with significantly reduced serum neutralizing antibodies and mucosal anti-SARS-CoV IgA in the aged host. This limited antibody response may be related to the dramatically reduced lung and peripheral B lymphocyte populations observed in the aged monkeys which is consistent with reports in elderly humans [39]. Despite the deficiencies observed in the aged adaptive responses, viral titers were only significantly higher in aged monkeys at day 1 post infection. This suggests that innate or other compensatory immune responses are sufficient for viral control in aged monkeys and serves to further support the minimal involvement of cytotoxic T cells and neutralizing antibodies in SARS-CoV clearance as demonstrated in mouse depletion models [36].

Taken together, our data indicate that systemic and mucosal immunity to SARS-CoV infection differs in the aged as compared to the juvenile host. This knowledge will be important to consider in the design of effective intervention strategies for SARS-CoV and potentially other respiratory infections. Our experimental results support future mechanistic studies to identify adjunctive strategies capable of overcoming the immune deficits of the aged airway mucosa, which may ultimately translate into novel approaches to enhance efficacy of vaccines against respiratory pathogens for the elderly population.

## Conclusions

In this study we found that viral titers in aged, as compared to juvenile monkeys were significantly higher early after infection, but levels became comparable in both age groups at later infection time points. We observed significant age and infection-dependent differences in both the systemic and mucosal immune compartments with more dramatic changes in cytokine levels and leukocyte frequencies in lung as compared to peripheral blood and tracheobronchial lymph nodes. In regards to the exacerbated SARS-CoV responses previously reported in aged nonhuman primates, we did not observe enhanced host immunity or pathology in our aged monkeys. Instead, we detected less inflammatory cytokines and total lung leukocytes, as well as reduced adaptive responses with increased age. Although our results are discrepant, both studies indicate that pulmonary immunity is intrinsically different in the aged as compared to the juvenile host, warranting further exploration given the important implications this has for vaccine and therapeutic design for the elderly.

## Methods

### Ethics statement

All procedures were conducted under protocols approved by the Institutional Animal Care and Use Committee (IACUC) at Lovelace Respiratory Research Institute (LRRI), all facilities were accredited by the Association for Assessment and Accreditation of Laboratory Animal Care International (AAALAC), and guidelines for nonhuman primates described in the Guide for the Care and Use of Laboratory Animals, National Research Council, were strictly adhered to.

### Nonhuman primates

The animals used in this study were adult African green monkeys obtained from the Barbados Primate Research Centre, Barbados, West Indies through Global Research Supply, LLC (Reno, NV), a Class B USDA licensed animal dealer. A permit to import the animals into New Mexico was acquired through the Fish and Wildlife Service by LRRI. The aged African green monkeys were determined to be ~10-20 yrs old while the remaining subjects were young adults (~2-6 years old). Additionally, two female African green monkeys of 17 yrs and 18 yrs were acquired from the Wake Forest University Primate Center (Medical Center Boulevard, Winston-Salem). As there is an estimated 3.2-fold difference for relating age between humans and rhesus macaques [26,27], the aged African green monkeys in our study are expected to represent approximately, 50 year old humans while the juvenile African green monkeys correspond to 6–12 year olds. The rhesus-to-human age conversion was used given that the estimated age conversion is not as well established for African green monkeys and there is 95% genetic similarity [40] between the two Old World monkey species belonging to the Super Family Cercopithecoidea. Randomization and group assignments for the aged monkey studies were performed with Provantis Integrated Preclinical Software (Instem Life Science Systems Ltd.). All monkeys were quarantined for 6 weeks prior to the study, during which they were tested for tuberculosis. Animals were given a subcutaneous microchip for identification and temperature measurements (IPTT-300 implantable temperature transponder and a WRS-6007 Handheld Wireless Reader System (Bio Medic Data Systems, Inc, Seaford, Delaware)). During the infectious portion of the study, animals were individually housed indoors in stainless steel cages with wire mesh bottoms. Temperature and humidity ranges were controlled along with 12 h light and dark cycles. Animals were also given environmental enrichment including toys twice per day. Tap water from the institutional watering system was available ad libitum and animals were fed twice a day Harlan Teklad Certified 20% Monkey Diet (4 to 6 biscuits/kg body weight) with bananas or apples for enrichment 3 times per week.

### SARS-CoV infection of African green monkeys

The SARS-CoV strain HKU-39849 was provided by Dr. Leo Poon (Department of Microbiology, University of Hong Kong, Hong Kong, China) and viral stocks were generated in Vero E6 cells. Experimental infection with 10^7^ plaque-forming units (PFU) of SARS-CoV instilled intranasally was conducted as previously reported [28]. Monkeys were examined by trained Laboratory Animal Technicians twice per day (at least 6 h apart) on each day of the study for parameters including appetite, appearance, activity, stool, posture, neurological signs, respiration, ocular discharge and nasal discharge. Onset of any abnormal clinical sign was documented and a score sheet completed each day beginning from the date the animal fell ill. Euthanasia to alleviate suffering was conducted based on the clinical observation score sheet and consultation with the Staff Veterinarian. Animals were sacrificed at 5 and 10 d.p.i. (n = 6 for juvenile and n = 5 for aged animals at each time point) by intravenous overdose of Euthasol® (Virbac AH, Inc., Fort Worth, TX). Virus-free cell culture medium was used to inoculate mock-infected controls (n = 6 for juveniles and n = 2 for aged). Some of the immunology, virology and pathology data for the juvenile animals were previously reported [28] but are included in this manuscript for comparison with aged monkeys.

### Plaque and neutralization assays

Virus titers were measured in plaque assays by applying serial dilutions of homogenized tissue supernatants onto Vero E6 cell monolayers as previously described [28]. To measure SARS-CoV neutralizing antibodies in serum, samples were serially diluted and incubated with 2,000 PFU/ml SARS-CoV overnight before inoculation onto Vero E6 cells. Titers are expressed as the reciprocal of the highest dilution at which the cytopathic effect was completely inhibited.

### Flow cytometry

Standardized collected lung tissue from the proximal portion of the right caudal lobe was processed into single-cell suspensions for flow cytometry. Of note, the samples for flow cytometric analysis and cytokine protein evaluation were acquired from adjacent tissue regions. Single cell suspensions were prepared as detailed in [28]. Briefly, mechanical and enzymatic digestion with Liberase (Roche, Pleasanton, CA) and DNAse (Sigma, St. Louis, MO) solution was performed prior to overlaying samples on a Percoll (Sigma) gradient for 20 min at 500 × g with no brake. Tracheobronchial lymph nodes were collected in a standardized manner in which the same region and size of tissue was collected from each animal. Single-cell suspensions were made by mechanical disruption. Peripheral blood mononuclear cells (PBMC) were isolated from heparinized blood with a Ficol-Hypaque (Sigma) gradient as previously reported [41]. All flow cytometric analysis was conducted on previously frozen PBMC, lung or lymph node leukocytes. The following antibodies were used in 4- or 6- color staining panels: CD3 (clone SDP34-2); CD8 (clone SK1); CD11c (clone SHCL-3); CD14 (clone M5E2); CD20 (clone L27); CD68 (clone KP1, Santa Cruz Biotechnologies, Dallas, TX); CD86 (clone 2331Fun1); Granzyme B (clone GB11, Invitrogen, Carlsbad CA); HLA-DR (clone L243); Ki67 (clone B56); CCR1 (clone 53504); CCR3 (clone 61828); CCR5 (clone 3A9); CCR6 (clone 11A9) and CCR7 (clone 150503, R&D Systems, Minneapolis, MN) all conjugated to fluorochromes FITC, PE, PerCP, PerCpCy5.5, APC, PECy7, Alexa647 or APCCy7 (BD Biosciences, San Jose, CA unless specified). For intracellular antigens, BD FACS lyse and permeabilization solutions were used according to the manufacturer’s instructions. CD4 was not assessed given the downregulation of this marker on African green monkey T lymphocytes [42-44]. Antibody-stained samples were fixed for 16 hr in 1% paraformaldehyde and 3% FBS in phosphate buffered saline. Sample data were acquired on a FACS-Calibur or FACS-Canto flow cytometer instrument (BD Biosciences) and data files analyzed utilizing FlowJo software 7.6.1 (TreeStar, Medford, OR). For all flow cytometric analysis, within one experiment, the gates applied to each sample were identical (See gating strategies in Additional file 1: Figure S1, Additional file 2: Figure S2 and Additional file 4: Figure S3). When appropriate and possible, similar gates were used across the two different experiments (juvenile and aged samples). However, based on changes to flow cytometer settings and fluorescence minus one controls, some gates were adjusted between the two experiments. Markers were chosen for characterization of specific leukocyte subsets in PBMC, lymph node, and lung based on previously published studies in humans or nonhuman primates [45-53].

### ELISAS and cytokine bead-based assays

Lung tissue was acquired in a standardized collection scheme in which a section of the proximal right caudal lobe was divided into two portions, one for homogenization to assess cytokines and antibodies and virus while lung leukocytes were isolated from the other portion for flow cytometry (see previous section). For homogenization, a volume of RPMI media (Gibco, Life Techologies, Grand Island, NY) equal to 10% of the lung tissue weight was added. SARS-CoV IgG and IgA antibodies were measured by ELISA detailed in [28]. Briefly, ELISA plates were coated with purified recombinant SARS-CoV S protein in carbonate-coating buffer (S protein NR-686 obtained through NIH Biodefense and Emerging Infections Research Resources Repository) and nonspecific binding blocked with PowerBlock (Biogenex, San Ramon CA). Starting at a 1:25 dilution, serially diluted lung tissue homogenate supernatants (clarified by centrifugation) were incubated on S-protein coated plates overnight at 37°C. An anti-monkey IgG or IgA HRP-conjugated antibody (KPL Inc., Gaithersburg, MD) was applied followed by substrate development with ABTS Microwell peroxidase substrate system (KPL) and absorbance reading at 405 nm using a Thermo Electron Corporation plate reader (Thermo Electron Corporation, Houston, TX). Data was acquired with Ascent software (Ascent Software, London, UK) and the ELISA antibody titer recorded for each sample was the reciprocal of the highest dilution in which the optical density (O.D.) reading for S-protein bound wells was at least two-fold higher than that of the nonfat milk control. The O.D. of the highest titer chosen also had to fall within the linear range of the serial dilutions.

Cytokine and chemokine protein was measured in lung tissue homogenates and serum using both human and nonhuman primate multiplex bead-based array kits (Millipore, Billerica, MA). The assays were performed according to the manufacturer’s instructions with an overnight incubation of the samples in antibody-immobilized beads. Data was collected on the Bio-Plex System (BioRad, Hercules, CA) and a weighted 5-parameter logistic curve-fitting method used to calculate the concentration of individual analytes. All measurements were performed in duplicate and data reported as pg/ml. CD40L was undetectable in serum of either age group and IL-5, TNF-alpha, IL-17, IL-2, CCL24 and CCL26 were below the level of detection in lung tissue homogenates.

### Histopathology

Sampling of tissues for histopathology was performed as previously reported [28] in a standardized manner with random assessment. All organs were fixed in 4% paraformaldehyde solution prior to embedding in paraffin. 5 μm thick tissue sections were stained with hematoxylin and eosin for examination microscopically by a board certified veterinary pathologist. Histologic lesions were graded for severity (0 = normal, 1 = minimal, 2 = mild, 3 = moderate, 4 = marked) and distribution, (focal, multifocal, diffuse).

### Statistical analysis

Infection and age differences were evaluated using a 2-way ANOVA with Bonferroni post-tests or two-tailed student T-tests, where appropriate. All data for statistical evaluation was log transformed and analyzed with GraphPad Prism 5.0 software (GraphPad, La Jolla, CA). A *p* value of 0.05 or less was considered statistically significant.

## Abbreviations

SARS-CoV: Severe acute respiratory corona virus; d.p.i.: Days post infection; PFU: Plaque-forming units; DC: Dendritic cell.

## Competing interests

The authors declare no financial or non-financial competing interests in the work presented in this study.

## Authors’ contributions

CCC data collection, analysis and interpretation of data, figure preparation, manuscript writing; ND data collection, analysis, manuscript editing; NF conception and design, analysis and interpretation of data; JBK data collection, analysis, manuscript editing; KO conception and design, study director, manuscript editing; JT data collection, data analysis, manuscript editing; JVW data collection, analysis, manuscript editing; FH board-certified pathologist, histopathology analysis, manuscript editing, KSH conception and design, interpretation of data, final approval of the manuscript. All authors read and approve the final manuscript.

## Supplementary Material

Additional file 1: Figure S1 Gating strategy for analysis of extracellular markers on B and T cells. The representative gating strategy for flow cytometric analysis of lung, lymph node and PBMC is illustrated based on PBMC from an aged monkey. First a lymphocyte gate based on forward and side scatter is applied with the percent frequency of total cells indicated in the plot (FSC/SSC). CD8 T cells were subsequently gated based on CD3 and CD8 expression (CD3 + CD8+). CD8 T cells were further evaluated for coexpression of naïve cell markers CCR7 and CD45RA (CCR7 + CD45RA+) or CCR5 expression. B lymphocytes were gated based on CD20 expression and absence of CD3 (CD3-CD20+). The percent frequencies of total gated lymphocytes based on the FSC/SSC gate are indicated in each plot. Click here for file

Additional file 2: Figure S2 Gating strategy for analysis of intracellular markers. The gating strategy for flow cytometric analysis of macrophages, dendritic cells and CD8 T cells in which intracellular markers were used is shown using a representative lung sample from an aged animal. Leukocytes were first gated on forward and side scatter with the percent frequency of total cells indicated in the plot (FSC/SSC). Cells were subsequently gated on CD68 and HLADR for macrophages and dendritic cells (DCs) (CD68/HLADR). Macrophages were defined as CD68 + HLADR + and were evaluated for expression of CD86. CD68 negative HLADR + cells were further gated on CD11c positive to define DCs (CD68-HLADR + CD11c+) which were also evaluated for CD86 expression. CD8 T cells were defined as CD3 + CD8+ and were assessed for expression of intracellular antigens, Ki67 and granzyme B. The percent frequencies of total gated leukocytes based on FSC/SSC are indicated in each plot. Click here for file

Additional file 3: Table S1. Relative frequency of leukocyte populations in the lung and lymph node during SARS-CoV infection. Click here for file

Additional file 4: Figure S3 Gating strategy for peripheral monocytes and dendritic cells. The gating strategy for monocytes and DCs in the peripheral blood is depicted using a representative aged PBMC sample. Cells are first gated based on forward and side scatter with the percent frequency of total cells indicated in the plot. The subsequent gate is for CD14 and HLADR expression in which monocytes are defined as CD14 + HLADR+. Monocytes are subsequently evaluated for CD86, CCR1 and CCR3 expression. CD14negative HLADR + cells are further gated on CD11c expression to define DCs (CD14-HLADR + CD11c+). CD86 and CCR6 expression was then evaluated on DCs. The percent frequencies of total gated leukocytes based on FSC/SSC are indicated in each plot. Click here for file
