# Supplementary figures and images for: Severe acute respiratory syndrome-coronavirus infection in aged nonhuman primates is associated with modulated pulmonary and systemic immune responses
Source: Immun Ageing. 2014 Mar 19;11:4. doi: 10.1186/1742-4933-11-4 (PMC3999990; doi:10.1186/1742-4933-11-4)

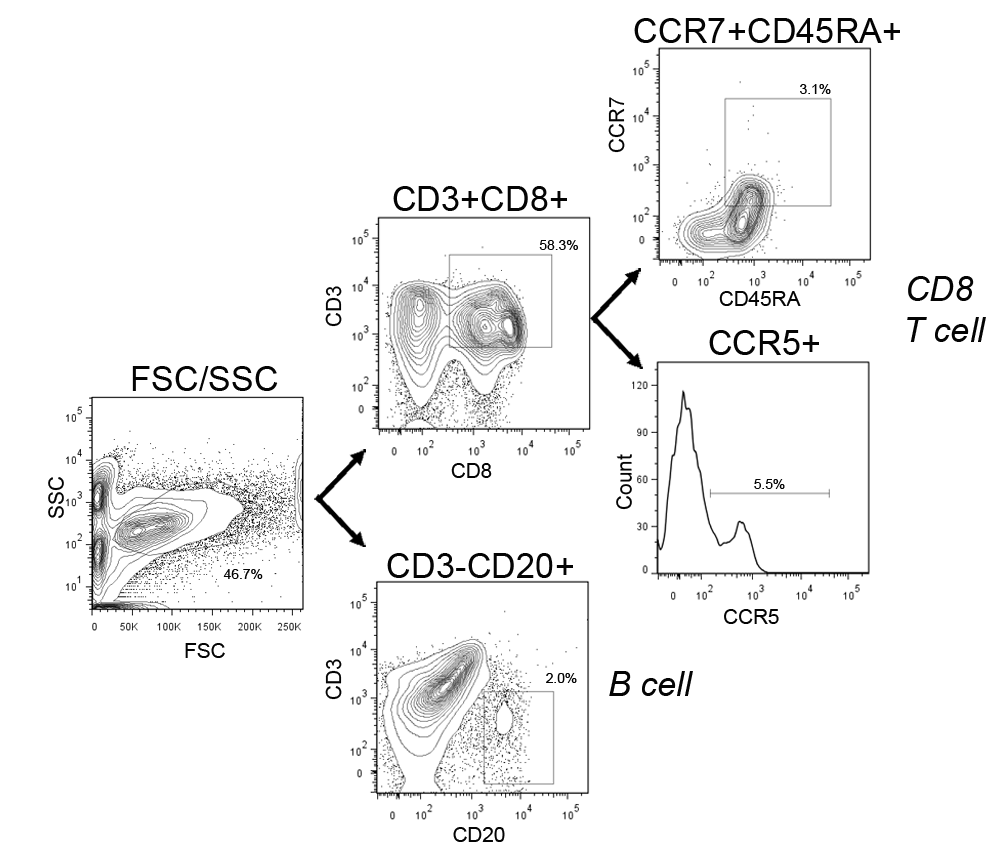

Supplement: Additional file 1: Figure S1 — Gating strategy for analysis of extracellular markers on B and T cells. The representative gating strategy for flow cytometric analysis of lung, lymph node and PBMC is illustrated based on PBMC from an aged monkey. First a lymphocyte gate based on forward and side scatter is applied with the percent frequency of total cells indicated in the plot (FSC/SSC). CD8 T cells were subsequently gated based on CD3 and CD8 expression (CD3 + CD8+). CD8 T cells were further evaluated for coexpression of naïve cell markers CCR7 and CD45RA (CCR7 + CD45RA+) or CCR5 expression. B lymphocytes were gated based on CD20 expression and absence of CD3 (CD3-CD20+). The percent frequencies of total gated lymphocytes based on the FSC/SSC gate are indicated in each plot. [file 1742-4933-11-4-S1.tiff]

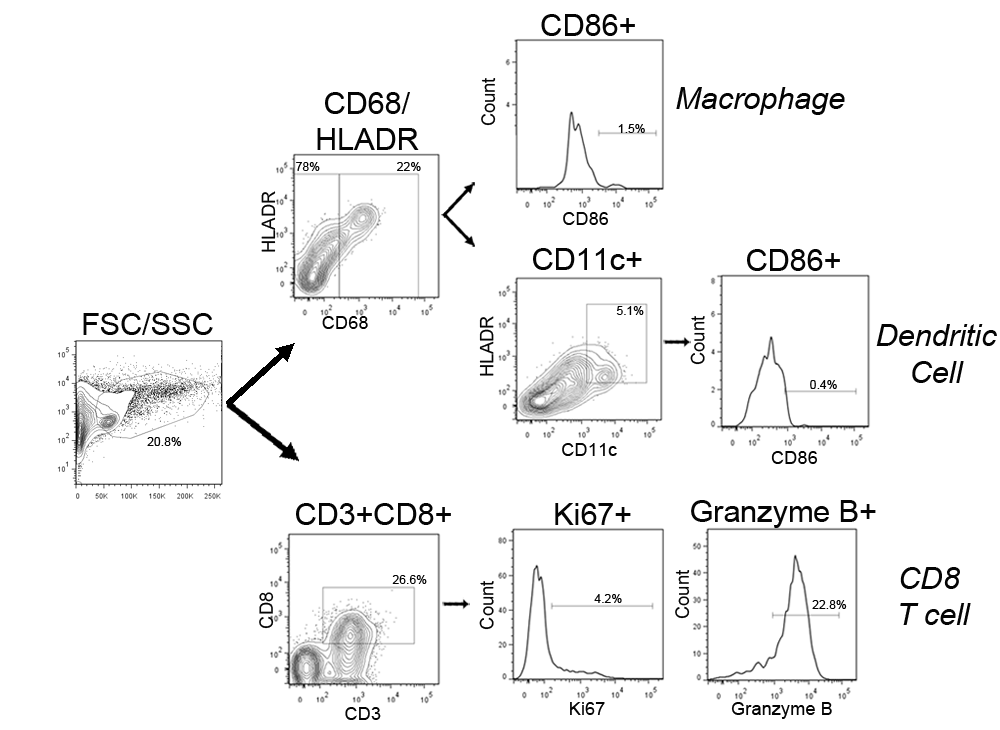

Supplement: Additional file 2: Figure S2 — Gating strategy for analysis of intracellular markers. The gating strategy for flow cytometric analysis of macrophages, dendritic cells and CD8 T cells in which intracellular markers were used is shown using a representative lung sample from an aged animal. Leukocytes were first gated on forward and side scatter with the percent frequency of total cells indicated in the plot (FSC/SSC). Cells were subsequently gated on CD68 and HLADR for macrophages and dendritic cells (DCs) (CD68/HLADR). Macrophages were defined as CD68 + HLADR + and were evaluated for expression of CD86. CD68 negative HLADR + cells were further gated on CD11c positive to define DCs (CD68-HLADR + CD11c+) which were also evaluated for CD86 expression. CD8 T cells were defined as CD3 + CD8+ and were assessed for expression of intracellular antigens, Ki67 and granzyme B. The percent frequencies of total gated leukocytes based on FSC/SSC are indicated in each plot. [file 1742-4933-11-4-S2.tiff]

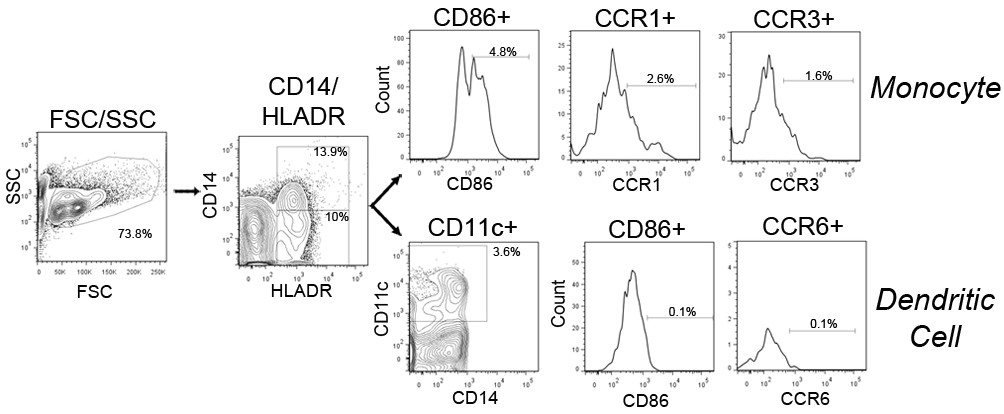

Supplement: Additional file 4: Figure S3 — Gating strategy for peripheral monocytes and dendritic cells. The gating strategy for monocytes and DCs in the peripheral blood is depicted using a representative aged PBMC sample. Cells are first gated based on forward and side scatter with the percent frequency of total cells indicated in the plot. The subsequent gate is for CD14 and HLADR expression in which monocytes are defined as CD14 + HLADR+. Monocytes are subsequently evaluated for CD86, CCR1 and CCR3 expression. CD14negative HLADR + cells are further gated on CD11c expression to define DCs (CD14-HLADR + CD11c+). CD86 and CCR6 expression was then evaluated on DCs. The percent frequencies of total gated leukocytes based on FSC/SSC are indicated in each plot. [file 1742-4933-11-4-S4.tiff]
