# Supplementary material for: Severe acute respiratory syndrome-coronavirus infection in aged nonhuman primates is associated with modulated pulmonary and systemic immune responses
Source: Immun Ageing. 2014 Mar 19;11:4. doi: 10.1186/1742-4933-11-4 (PMC3999990; doi:10.1186/1742-4933-11-4)
Supplement: Additional file 3: Table S1. — Relative frequency of leukocyte populations in the lung and lymph node during SARS-CoV infection. [file 1742-4933-11-4-S3.docx]

| **Supporting Table 1. Relative frequency of leukocyte populations in the lung and lymph node during SARS-CoV infection** | | | | | | | | |
| --- | --- | --- | --- | --- | --- | --- | --- | --- |
|  |  |  |  |  |  |  |  |  |
|  | Frequency of cells^a^ | |  |  |  |  |  |  |
| *Lung* | Mock | |  | Day 5 | |  | Day 10 | |
|  | Juvenile | Aged |  | Juvenile | Aged |  | Juvenile | Aged |
| Macrophages | 41.7 + 11.6 | 18.0 + 3.1 |  | 45.4 + 6.7 | 32.1 + 4.4 |  | 48.1 + 8.9 | 15.3 + 5.5* |
| DCs | 10.4 + 1.6 | 4.8 + 1.9 |  | 11.5 + 2.2 | 5.5 + 0.6* |  | 10.7 + 1.4 | 3.6 + 0.9* |
| CD8 T cells | 35.4 + 11.0 | 18.8 + 3.2 |  | 44.0 + 5.3 | 19.5 + 2.0* |  | 33.8 + 6.3 | 24.1 + 5.1 |
| B cells | 1.6 + 0.4 | 0.7 + 0.1 |  | 2.9 + 0.9 | 0.3 + 0.1 |  | 4.1 + 0.7 | 0.4 + 0.1* |
| *Lymph node* | Mock | |  | Day 5 | |  | Day 10 | |
|  | Juvenile | Aged |  | Juvenile | Aged |  | Juvenile | Aged |
| CD8 T cells | 32.4 + 3.5 | 20.2 + 0.9 |  | 38.6 + 4.0 | 26.8 + 3.5 |  | 30.9 + 1.9 | 26.8 + 1.1 |
| B cells | 14.8 + 2.4 | 14.1 + 5.3 |  | 20.3 + 1.5 | 16.4 + 6.3 |  | 21.1 + 2.1 | 19.8 + 2.3 |
| ^a^Average frequency of macrophages (CD68+HLADR+), DCs (CD11c+CD68-HLADR+), CD8 T cells (CD3+CD8+), B cells (CD20+) of total lung or lymph node leukocytes. Gating strategies shown in Supplementary Figures 1 and 2. Asterisks indicate values that are significantly different (*p*<0.05) between the two age groups by unpaired student T-test. | | | | | | | | |
|  |  |  |  |  |  |  |  |  |
|  |  |  |  |  |  |  |  |  |
